# The life-changing magic of doing a sabbatical

**DOI:** 10.1038/s44319-023-00042-0

**Published:** 2024-01-02

**Authors:** K Heran Darwin

**Affiliations:** New York University Grossman School of Medicine, New York, NY USA

**Keywords:** Careers, Methods & Resources, Microbiology, Virology & Host Pathogen Interaction

## Abstract

Why do so many academics ignore an amazing perk of our job?

**Figure:**
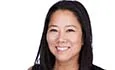

I once happened to mention to an editor at a conference that I was about to go on sabbatical, which resulted in a brief commentary on sabbaticals (Clevers et al, 2015). By definition, a sabbatical is a break from work and many academic institutions permit their faculty to visit another research institute for an extended period after they have been awarded tenure. Remarkably, they often cover 100% of the salary during this time, which can save precious grant dollars for research. For most of us, it does not mean a complete abandonment of the lab or office though, but rather an opportunity to learn something new.

A sabbatical sounded like a fantastic idea after we were hit in late 2012 by superstorm Sandy, which greatly affected New York City. The turmoil of four lab moves after the storm convinced me that I needed a change of scenery, even if for just a few weeks. But it does not have to take a natural disaster to inspire a sabbatical. Raphael Valdivia at Duke University explained that “I needed a reboot, change of environment, an opportunity to try something new and especially have time and space to THINK. I wanted to rekindle that enthusiasm from my postdoc days when science was just fun.” Randy Hampton at UCSD pointed out that “First, [sabbatical] is the rarest of opportunities in the general workspace. There are almost no other occupational niches that would even consider this kind of subsidized freedom. For a long time, I had hesitation about leaving my lab for that long a time, but the freedom that the group got, and perhaps the breathing room, was a plus. Second, I explored some things that really interested me, not for direct application to the work we do, but things that I was very curious about and thought would edify me.” He added, “DO IT.”

The first and perhaps most important decision is with whom to do a sabbatical. Raphael suggested, “Get out of town and try something completely different. If possible, get into the lab and do experiments. Chat with students, socialize with new colleagues, explore your new environment.” For me as a card-carrying microbiologist, the toothpick is my sword, but I needed to start caring about the beleaguered host that struggles to defeat my microbe of interest, *Mycobacterium tuberculosis*, a.k.a. the honey badger of pathogens (Darwin, 2021). The immune system had always intimidated me: a Byzantine cellular and molecular network that makes me want to poke my eyes out whenever I try to understand its workings. Nevertheless, I overcame this misguided desire and reached out to Russell Vance at UC Berkeley, who wanted to grow a TB program in his lab. His research focuses on how we sense and respond to invading microbes, which is a primary function of the innate immune system. While most humans successfully defend against constant attacks by infectious agents, many microbes have evolved to either evade or persist in the face of whatever the immune system throws at them. Given that *M. tuberculosis* is among these pathogens, Russell was eager to gain a better understanding of how some hosts suppress its growth better than others, but he had to convince his team to study a new and difficult organism in a lab that had little to no experience working with it. The timing of our mutual interests worked out perfectly because Russell had set up a “staybattical”, a break from his administrative and teaching duties, to gain more experience with *M. tuberculosis*.

I taught Russell the basics: while making agar plates and setting up aerosol infections might seem simple, there are many tips and tricks that are often not intuitive in typed protocols. I believe having this new knowledge allowed him to more confidently advocate for *M. tuberculosis* experiments in his lab. For me, it was the immersive experience that allowed me to better appreciate the many ways how hosts detect and resist infection. I also have a much better appreciation for the challenges of trying to understand complex molecular mechanisms in multicellular organisms.

A lot of planning goes into a sabbatical, which can prevent some academics from taking one. At NYU Grossman School of Medicine, we are required to apply for permission to take a sabbatical up to a year in advance. In addition, if you have extramural funding, granting institutions need to be made aware why salary is not being drawn down from your accounts. Another significant challenge is choosing a location, which can dramatically affect how much it will cost. Most folks cannot charge their institutions or grants for housing and if your host does not have a spare room, you will have to find a place to live. Some institutions have housing or offer sabbatical home swaps, so it is worth asking if there is a website that lists available housing at the host institution. I eventually opted for a vacation rental site which I paid for out of my own pocket and Russell helped check out listings before I committed. I also didn’t have a car, so it was important for me to be able to get around on foot or public transportation if I could not bum a ride from someone on campus.

Speaking of extramural funding, Raphael recommended taking a sabbatical when you are not under pressure to submit grant applications, if possible. However, my colleague Greg David pointed out that even when there was pressure to seek funding, “it was beneficial to not have to think about writing grants during the sabbatical, as it allowed me to feel fully immersed in this new lab culture and research. Writing grants can be very isolating, and the goal of the sabbatical was to learn by being an integral member of a new team. But then, as soon as I went back to NYUGSM [NYU Grossman School of Medicine], I could write a grant based on the new research avenues explored during the sabbatical.”

Taking a sabbatical also doesn’t mean ditching the lab. I scheduled weekly video meetings to keep projects moving smoothly. I also returned home every few weeks to make sure my lab had not burned down. I found these regular visits essential to keep motivation up and projects moving forward.

There were many benefits I gained from my time in Berkeley. Scientifically, it changed the way I think about how the host defends against invading pathogens—I used to anthropomorphize bacteria as intentional in their motivations to harm us, but I now appreciate that exuberant inflammation is often an outlier outcome of infection, and that our immune system usually fights off infection without us even knowing it. I also learned about how metabolism dramatically changes in the host during infection, and how these changes likely contribute to infection outcome. Since my sabbatical ended in early 2016, the Vance lab has published several papers that have revealed critical new insights into how the field thinks about TB infections, and I am grateful for being a small part of these contributions. Moreover, conversations I had with faculty at Berkeley inspired a joyous collaboration with Sarah Stanley’s lab, which has taken us both in exciting new directions, in addition to sparking a marvelous friendship.

I have continued to benefit in other ways, notably from the new friends I made, including students and post-docs who have continued to pursue research careers all over the country. These new colleagues have provided helpful scientific advice, but more importantly, it has been a pleasure to watch them develop their own programs. I was also lucky to recruit a ridiculously talented student from Russell’s lab for her post-doctoral studies.

There were also unexpected benefits. As corny as it sounds, absence does make the heart grow fonder. On my return to New York, I was happy to get back home and to the lab, a sentiment echoed by Randy. It was reassuring to know that while the lab managed to do without me, they still needed me. Lab members seemed to be truly happy to have me back, and it was exhilarating to be in the thick of discovery again. If that is not life-changing magic, I do not know what is.
